# Influence of Occlusal Cusp Inclination and Restoration Thickness on the Biomechanical Performance of Lithium Disilicate Molar Endocrowns: A 3D Finite Element Analysis

**DOI:** 10.1055/s-0045-1810442

**Published:** 2025-08-07

**Authors:** Layla Hassouneh, Nawar Naguib Nawar, Mohammad Atieh, Dilek Yigit, Venkateshbabu Nagendrababu, Ove A. Peters, Manal Matoug-Elwerfelli

**Affiliations:** 1 Department of Conservative Dentistry, Faculty of Dentistry, Jordan University of Science and Technology, Irbid, Jordan; 2 Department of Endodontics, Faculty of Dentistry, The British University in Egypt, Cairo, Egypt; 3 Department of Pre-Clinical Oral Sciences, College of Dental Medicine, QU Health, Qatar University, Doha, Qatar; 4 Department of Restorative Dentistry, College of Dental Medicine, University of Sharjah, Sharjah, United Arab Emirates; 5 School of Dentistry, University of Queensland, Brisbane, Australia

**Keywords:** endocrowns, finite element analysis, lithium disilicate, occlusal design, root canal treated teeth, stress distribution

## Abstract

**Objectives:**

This study used finite element analysis to investigate the effect of occlusal cusp inclination and restoration thickness of endocrowns on the stress distribution and biomechanical performance.

**Materials and Methods:**

A total of six models of a mandibular first molar representing two different heights of remaining tooth structure above the cemento-enamel junction (1.5 and 3 mm), each with three different buccal cusps inclination angles (original, 10 degrees, and 20 degrees increase in cusp inclination angles) were generated. Models were designated as: 1.5/original, 1.5/10 degrees, 1.5/20 degrees, 3/original, 3/10 degrees, and 3/20 degrees. All models were subjected to an oblique load of 400 N. The maximum principal stress (MPS), maximum shear stress (MSS) at the cement interface, and factor of safety (FoS) were calculated.

**Results:**

Increasing the cuspal inclination by 10 degrees in models with a 1.5-mm remaining tooth structure, resulted in a 20% reduction of the MPS in the dentine (27.2 MPa), in comparison with original cuspal inclines (33.9 MPa). However, increasing the cusp inclination in model 1.5/20 degrees resulted in a comparable dentinal stress reduction (17%, 28 MPa) accompanied with an increase in the MSS at the cement interfaces (26.8 MPa), in comparison with 1.5/original (18 MPa). On the other hand, increasing the cusp inclination angle in models 3/10 degrees and 3/20 degrees led to a reduction in MPS within the dentine by 1 and 2%, respectively, while causing an increase in the MSS at the cement interfaces (16.4 and 16.0 MPa, respectively), in comparison with 3/original (11 MPa). Models 1.5/original and 3/original reported the minimum FoS values (3.10 and 3.38, respectively), while model 1.5/10 degrees reported the highest FoS value (3.86).

**Conclusion:**

Within the limitations of the current study, cusp inclination adjustments up to 10 degrees may enhance stress distribution in endocrown-restored molars with limited coronal structure.

## Introduction

The restoration of endodontically treated teeth (ETT) with extensive loss of structure continues to be a challenging procedure in restorative dentistry as they are more prone to fractures than vital teeth. 1 2 First introduced in the late 1990s, endocrowns have recently gained renewed attention as an alternative and more conservative option to restore ETT. 3 4

The endocrown restoration combines multiple design features to enhance biomechanical performance and retention. An occlusal onlay-like preparation is performed to achieve cuspal coverage and protection, helping to reduce the risk of fracture by redistributing occlusal forces away from the weakened tooth structure. 4 The intraradicular extension of the endocrown material into the endodontic access cavity increases the surface area available for bonding, thereby enhancing the overall retention of the restoration. 5 Moreover, preserving enamel at the cavosurface margin provides a reliable substrate for adhesive bonding. 6

Several studies investigated the ideal restorative material to fabricate endocrowns, reporting the use of monolithic computer-aided design and different computer-aided manufacturing materials. 5 7 8 Among these materials, lithium disilicate glass ceramics (LDGC) has been recommended, mainly as they possess high esthetic and high adhesion values. 9 10

The long-term clinical performance of indirect restorations depends, among other factors, on the physicomechanical properties of the prosthetic materials, as well as the preparation design parameters and anatomical features of the restoration. 11 12 Clinical design parameters such as margin geometry, preparation height, thickness, and cusp inclination significantly affect the restoration's performance and fracture behavior. 12 Steep cusp inclines have been of concern as one of the predisposing factors for the incidence of tooth fracture in posterior dentition. 13 Although the restoration design could be influenced by the clinical requirements, several adjustments of the cusp inclination and anatomic features strongly depends on the skills and preferences of the dental clinician or technician. 14

During clinical function, the restoration design and volume can also affect the ability of the restorative material to dissipate masticatory loads and the material's mechanical response during stress concentration. 15 16 Due to the larger material thickness in endocrown designs compared to conventional crowns, the stiffness of the whole restoration increases and the tension is reduced at the restoration–cement interfaces. In such cases, crack formation normally starts from the occlusal loading surface and the stress will be affected by the occlusal complexity of the restorations. 7 Although previous studies investigated the effect of different preparation designs of endocrown-restored teeth on fracture resistance, 2 investigations of the complex occlusal geometry design on the stress distribution and failure probability of all-ceramic LDGC endocrowns have not been reported.

Simulation studies, such as finite element analysis (FEA), are still valuable in endodontic research. They provide useful insights in cases where conducting randomized clinical trials is not feasible. 17 FEA-based studies are well accepted when investigating various biomechanical properties that can be difficult to assess under *in vitro* or *in vivo* conditions. 18 19 Therefore, the aim of this FEA study was to investigate the influence of the endocrown restoration thickness and cusp inclination angle, and a combination of these effects on the biomechanical behavior of LDGC molar endocrowns. The null hypothesis was that the restoration thickness and cusp inclination angle would not significantly affect the stress concentration of the LDGC endocrowns on molars.

## Material and Methods

### Sample Selection and FEA Model Generation

An intact mature human mandibular first molar was scanned with a resolution of 27 μm, using an Inveon Multimodality single photon emission computed tomography (CT) scanner (Siemens Preclinical Solutions, Knoxville, Tennessee, United States).

**Fig. 1:**
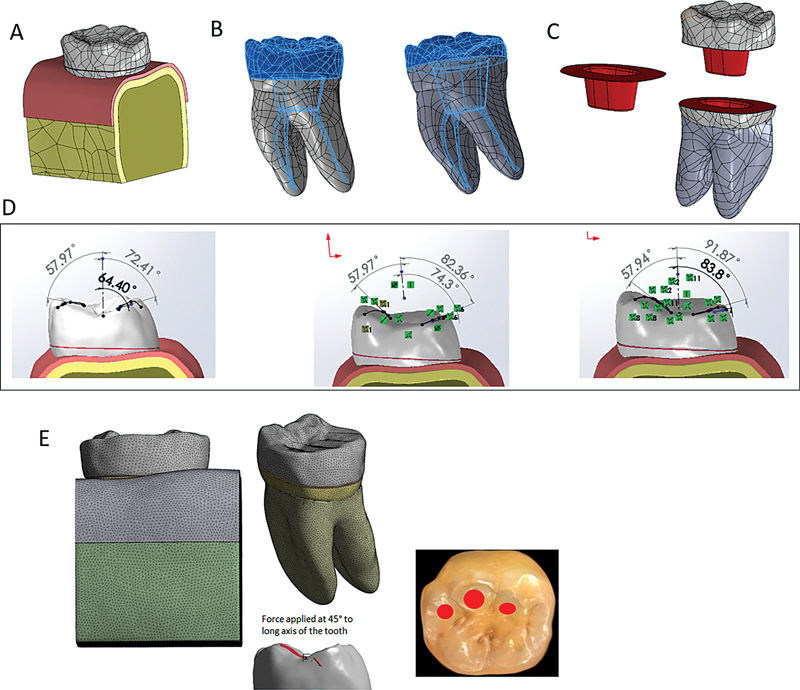
Simulated finite element analysis models, meshing pattern and loading conditions. ( **A** ) Illustration of the three-dimensional (3D) model composed of restored tooth, surrounding bone, and gingival tissue. ( **B** ) Illustration of the extension of endocrown central core into the endodontic access cavity and the tooth sectioning level above the cemento-enamel junction (CEJ); 1.5 mm (left) and 3 mm (right). ( **C** ) Exploded view of the endocrown, cement layer, and remaining tooth structure sectioned at 1.5 mm from the CEJ. ( **D** ) Illustration of cusp angle modification, original (left), 10 degrees increase in cusp inclination angle (center), 20 degrees increase in cusp inclination angle (right). The cusp angle was defined as the angle between the triangular ridge of the cusp and the vertical reference plane (parallel to long axis of tooth). ( **E** ) Illustration of mesh view of the entire model (left), restored tooth (upper right), and direction of force application (red arrow) in relation to the long axis of the tooth and location of force application (red areas lower right). The number of total nodes and elements in experimental models varied from 2036381 to 2065818 nodes, and 1380829 to 1396308 elements.

The scanned tooth had a typical occlusal anatomy with five distinct cusps of sufficient cusp inclination ( Fig. 1A ) as well as typical radicular anatomy with two mature roots enclosing three root canals. 20 Materialize interactive medical image control system (MIMICS version 21; Materialise, Leuven, Belgium) was used to reconstruct the Digital Imaging and Communications in Medicine images obtained. The software was used to identify enamel and dentine, form the masks, and grow threshold regions automatically. Regions were visually confirmed to avoid discrepancies before the three-dimensional (3D) model was produced. The periodontal ligaments and the surrounding bone investment were finally assembled with the tooth using SolidWorks (Dassault Systemes, Paris, France). Geomagic Design (3D Systems, Rock Hill, South Carolina, United States) was used to confirm the absence of exceptionally sharp edges or contact lines.

### Tooth Preparation and Endocrown Design

Two models were replicated from the original sound tooth model. The crown of the first model was sectioned at 1.5 mm above the cemento-enamel junction (CEJ), while the crown of the second model was sectioned at 3 mm above the CEJ ( Fig. 1A and B ). The access cavity walls were prepared with 8 degrees wall inclination angle, 21 and the root canals were symmetrically enlarged around their long axes to simulate radicular shaping before being filled with simulated gutta-percha. The utilized endocrown design included a butt joint occlusal preparation at the level of tooth sectioning and a central retention core extending from the level of the butt joint to the base of the access cavity. The occlusal portion of the endocrown corresponded to the original occlusal anatomy of the tooth before sectioning ( Fig. 1B and C ). The cement interface between the prepared tooth and endocrown was simulated with a thin layer of average thickness of 80 μm ( Fig. 1C ). 8

### Cusp Inclination Angle

For both designed models (sectioned at 1.5 and 3 mm above the CEJ), three cusp inclination angles for the buccal cusps (mesiobuccal, distobuccal, and distal cusp) were designed: original cuspal inclinations, 10 degrees increase in cusp inclination angle, and 20 degrees increase in cusp inclination angle ( Fig. 1D ). A total of six geometrical models were generated and designated as: 1.5/original, 1.5/10 degrees, 1.5/20 degrees, 3/original, 3/10 degrees, and 3/20 degrees. Endocrown elements were coupled to the material properties of LDGC while the cement layer elements were coupled to the material properties of resin cement (Variolink II, Ivoclar Vivadent, Schaan, Liechtenstein) ( Fig. 1A – C ).

### Meshing and Set Material Properties

The geometrical models described above were imported to the FEA software, ANSYS 19.3 software (ANSYS, Canonsburg, Pennsylvania, United States) for simulation. Parabolic tetrahedral solid elements were used in the meshing design of all experimental models. The meshes were generated through a convergence test of 10% strain energy and displacement variation. The mesh was checked for element quality and refined in the areas of interest ( Fig. 1E ). The mesh convergence test with total nodes and elements in each test model was as follows:

Test 1: 484523 and 347219 nodes and elements, respectively.
Test 2: 598862 and 427118 nodes and elements, respectively.
Test 3: 720209 and 541828 nodes and elements, respectively.

The final number of total elements and nodes in the experimental models varied between 541828 to 582435 elements and 720209 to 789504 nodes.

The simulated materials used in this study were considered linear, elastic, homogenous, and isotropic. Ideal adherence was assumed between structures. The applied material properties were based on previous FEA studies as listed in Table 1 . 18 22 23

**Table 1:** Mechanical properties adopted for simulated tooth tissue and restorative materials

Material/structure	Young's modulus (GPa)	Poisson's ratio
Enamel	84.1	0.33
Dentine	18.6	0.31
Resin cement	8.3	0.35
Lithium disilicate glass crown	95	0.3
Periodontal ligament	0.00427	0.45
Cortical bone	13.7	0.3
Cancellous bone	1.37	0.3
Gutta-percha	0.14	0.45

### Load and External Conditions

To simulate clinical masticatory conditions, all models were subjected to an oblique load of 400 N applied with an angle of 45 degrees to the longitudinal tooth axis ( Fig. 1E ). 24 25 The loading was applied at three defined points, specifically on the mid-lingual facing inclines of the mesiobuccal, distobuccal, and distal cusps, as illustrated in Fig. 1E . Movement restriction at the distal, mesial, and bottom surfaces of the bone were applied.

### Model Validation and Verification

The 3D mathematical model was directly validated against the natural tooth using the method detailed by a previous study. 26 Briefly, high-fusion wax (Galileo; Talladium Inc, Valencia, California, United States) was used to simulate the periodontal ligament, while a 3D-printed plastic block was employed to replicate the surrounding bone. 26 Next, the assembly was loaded three times by a universal testing machine and the displacement was registered. The results were compared to the FEA simulation predictions and a 5% maximum margin of error was deemed sufficient to validate the model.

The mesh design, solver settings, and boundary conditions were evaluated against established protocols in dental biomechanics literature. The finite element mesh was modeled based on validated approaches that approximate the complex geometries of the dental tissues and surrounding bone. Mesh convergence was performed as prescribed above. Material properties were derived from the most recent peer-reviewed studies, and the boundary conditions were reviewed to reflect realistic constraints such as those imposed by neighboring teeth. Verification procedures ensured consistency and the internal validity of the model.

### Data Analysis

The maximum principal stress (MPS) was analyzed in the following areas of interest; the remaining dentine and enamel tooth structure, endocrowns, and resin cement. Stress distributions in different structures were presented to visualize the overall mechanical behavior. The maximum shear stress (MSS) values at the interfaces of the resin cement layer were analyzed. 27 The factor of safety (FoS) theory was used to assess the safety of the models based on the Mohr–Coulomb failure criterion at the endocrown and remaining dentine tooth structure (higher FoS values indicate greater safety and stability under load). The dentine ultimate tensile strength (UTS) and ultimate compressive strength (UCS) are 105 and 298 MPa, respectively. 18 The Mohr–Coulomb stress ratio (ς _MC_ ) and FoS were calculated as the following:





where ς _max_ is the maximum tensile principal stress, and ς _min_ is the minimum compressive principal stress.

## Results

**Fig. 2:**
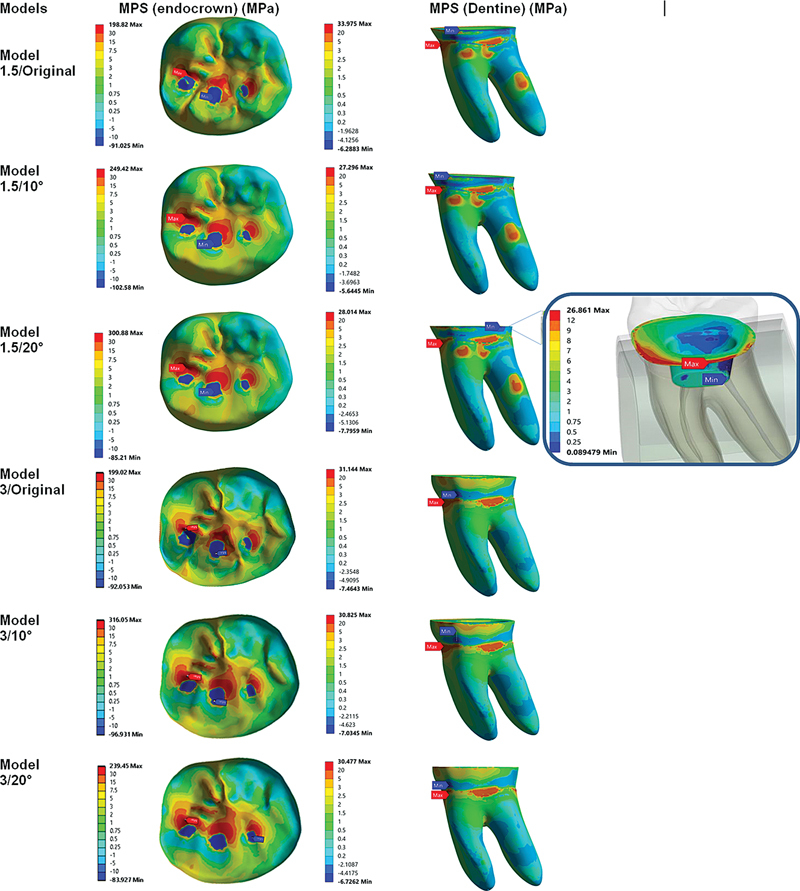
Graphical distribution of the maximum principal stress (MPS) values at the endocrown restoration and remaining dentine tooth structure of all six models under 400 N oblique loading force. Magnified image, to the far right, illustrates the maximum shear stress (MSS) at the cement layer interface.

The MPS, MSS, and the FoS values are listed in Table 2 . A visual representation is provided in Fig. 2 showing MPS values and distribution patterns computed for the six FEA models.

**Table 2:** The maximum principal stress, maximum shear stress at cement interfaces, and the minimum factor of safety for dentine of all models under 400 N oblique load

Models	Max. principal stress (MPa)						Max. shear stress at cement interfaces (Mpa)	Min. factor of safety for dentine
	Dentine		Enamel		Endocrown			
	Tension	Compression	Tension	Compression	Tension	Compression		
Model 1.5/original	33.975	–6.288	81.92	–106.46	198.82	–91.025	18.466	3.1052
Model 1.5/10 degrees	27.296	–5.6445	71.765	–101.43	249.42	–102.58	17.597	3.865
Model 1.5/20 degrees	28.014	–7.7959	75.367	–98.06	300.88	–85.21	26.86	3.766
Model 3/original	31.114	–7.464	92.82	–101.86	199.02	–92.05	11.19	3.3875
Model 3/10 degrees	30.825	–7.0345	91.433	–99.682	316.05	–96.931	16.429	3.422
Model 3/20 degrees	30.477	–6.7262	90.143	–97.645	239.45	–83.927	16.097	3.4617

### Models with 1.5 mm Remaining Tooth Structure Above the CEJ

In this group, model 1.5/original resulted in the highest MPS at the remaining dentine (34 MPa) and enamel (82 MPa), followed by 1.5/20 degrees with MPS values of 28 MPa at the remaining dentine and 75 MPa at enamel. Finally, model 1.5/10 degrees registered MPS values of 27 MPa at the remaining dentine and 72 MPa at enamel.

Within the endocrown material, the MPS values were highest for model 1.5/20 degrees (300 MPa), followed by model 1.5/10 degrees (250 MPa), and least with model 1.5/original (199 MPa).

At the cement interface, MSS was similar in models 1.5/original and 1.5/10 degrees (18 MPa), while a higher value was reported in model 1.5/20 degrees (26 MPa).

### Models with 3 mm Remaining Tooth Structure Above the CEJ

In this group, MPS values at the remaining dentine were similar across all three models (∼31 MPa). Enamel MPS values ranged from 90 to 93 MPa.

However, the endocrown material exhibited greater variation: model 3/10 degrees showed the highest MPS (316 MPa), followed by 3/20 degrees (239 MPa), and 3/original (199 MPa).

At the cement interface, MSS was similar in models 3/10 degrees and 3/20 degrees (16 MPa), while a lower value was reported in model 3/original (11 MPa).

Regarding the FoS values across all the six tested models, the minimum value was reported in model 1.5/original (3.10). For the remaining models, FoS ranged from 3.38 to 3.86, with model 1.5m/10 degrees reporting the highest FoS value (3.86).

### Stress Distribution Patterns

There were no differences in MPS distribution patterns among the three models in each group (1.5 and 3 mm above the CEJ). However, a comparison of dentine stress patterns between the two groups reveals some differences. In the 3-mm models, areas of elevated stress (red zones) were primarily located at the cervical region ( Fig. 2 ). In contrast, the 1.5-mm models demonstrated a wider extension of high stress areas (red zones), among the cervical area and the mid-root region ( Fig. 2 ). Nonetheless, all models consistently exhibited the highest stress concentration at the cervical region.

## Discussion

FEA-based studies are valuable in assessing the biomechanical performance of complex clinical scenarios and are extensively used in endodontics. 18 19 22 The clinical performance and failure of a given material or dental procedure are significantly influenced by the knowledge of stress concentration and distribution patterns. 28 Therefore, this study utilized a FEA design to investigate the influence of the complex occlusal geometry, namely, endocrown thickness, cusp inclination angle, and a combination of these effects on the biomechanical behavior of all-ceramic molar endocrowns.

Overall, it was noted that increasing the cusp inclination by 10 degrees in endocrown models with a 1.5-mm tooth structure above the CEJ (model 1.5/10 degrees), resulted in a 20% stress reduction of the MPS in the remaining dentine. However, when the cusp inclination was increased by 20 degrees (model 1.5/20 degrees), the resultant comparable dentinal stress reduction (17%) is accompanied by an increase in the MSS at the cement interfaces. On the other hand, increasing the cusp inclination angle in the endocrown models with a 3-mm height above the CEJ (models 3/10 degrees and 3/20 degrees) by 10 and 20 degrees resulted in minimal MPS reduction in the remaining dentine of 1 and 2%, respectively, while causing an increase in the MSS at the cement interfaces. Hence, the null hypothesis was rejected. Occlusal geometry impacted the magnitude of stress generated upon loading. Also, the height of the remaining tooth structure, which dictates the vertical dimension of the restoration, affects how these variables manifest in the biomechanical behavior of the assembly.

Due to the complex anatomy of molars, the complex interaction with surrounding supporting tissues and the wide range of bite forces (10–1000 N) among patients, evaluating stresses within teeth under masticatory loading is extremely challenging. 29 To establish an average posterior bite force value, we applied a force of 400 N, ensuring that the applied force is within a realistic and accepted range of posterior teeth. 30 The oblique direction of force application was chosen to combine the multidirectional nature of occlusal forces, where contact rarely occurs in a purely vertical direction. 31 32 Oblique loading replicate the worst-case clinical scenario, where unfavorable loading vectors may increase the risk of debonding or fracture and introduces shear and tensile stress components, which are more critical for evaluating failure risks. By doing so, the model's biomechanical response is tested under more demanding conditions, thereby enhancing the predictive value and safety margin of the restoration design being evaluated. 33 34

Stress distribution patterns within dentine do not seem to vary with the variation of the cuspal inclination when models with the same height of remaining tooth structure (1.5 or 3 mm) are compared. This can probably be explained by the fact that each triplet shared one restorative constant: the cement's volume, distribution, and layer thickness. Many studies have shown that interfaces hinder smooth force transition, 21 35 so it seems plausible that given the limited thickness of the cement between its two wide interfaces, occlusal forces passing through the endocrown thickness and the cement layer would reach dentine in a similar distribution despite differences in the magnitude among models. However, it should be noted that a uniform 80 μm cement layer was used in the present study for standardization, though this does not reflect clinical variability. 36 Micro-CT analysis has demonstrated that cement thickness is often greater in pulpal and cervical areas, which may affect stress distribution and crack initiation. 5 Although such modeling simplifications are common in FEA studies, future research should explore the impact of nonuniform cement layers on endocrown performance.

Although stress distribution patterns appeared similar across the 1.5 and 3 mm models, the consistent concentration of peak stresses in the cervical region suggests a higher risk of structural failure in this area, reinforcing the need for effective cervical support in clinical restorations. 7 On the other hand, the stress distribution patterns on the external surface of the root are different when dentinal stress is compared between any two models of the same cuspal inclination but different heights of the remaining tooth structure (1.5 vs. 3 mm). When this observation is put along the close range of dentine MPS values, it supports the finding reported by previous studies that dentine has the capacity to disperse stresses over a larger surface area, which brings down its magnitude. 26

Our findings also showed that the FoS seems to increase with increasing the cuspal inclination for models with the same vertical height of the shoulder. This can be attributed to the difference in force alignment as increasing the vertical cusp inclination angle brings the occlusal surface closer to a more favorable position to accommodate the load. The increase in FoS values was reported in the model with less remaining dental structure (i.e., larger restoration). In general, a FoS value greater than 1 suggests the model is safe, while a value less than 1 indicates failure. However, a FoS of 2 or more is commonly considered desirable in dental restorative applications to account for fatigue, stress concentrations, and patient variability. 37 38 Values above 3, as observed in the current study, suggest a substantial margin of safety, indicating a robust design capable of withstanding variable clinical loading conditions.

On the other hand, the 20-degree increase risked earlier debonding as the shear stresses within the cement layer increased to beyond or at least within the ranges of the maximum reported shear stresses a resin cement can take. 6 Debonding of the cement in this scenario would not be regarded as a catastrophic failure, as the little remaining tooth structure is uncompromised and allows for another restoration. Further investigations are needed to determine whether or not increasing the cuspal inclination (within the range permitted by the patient's occlusion) can be considered a safeguard with clinical scenarios requiring extensive restorations and in premolars where the small occlusal table, the wedging nature of the occlusion, and the larger length-to-width ratio compared to molars increase the risk on unrestorable failures. 7 Well-planned clinical studies are needed to confirm or refute the findings of this study.

The study limitations are mostly dictated by shortcomings of current FEA capabilities. FEA simulations assume the ideal execution of the design and restoration production, which eliminates the effect of human error, variations in cement film thickness, and the impact of thermocycling. 39 While applying standardized static loading enhances model comparability and isolates cusp inclination and remaining tooth structure effects, it does not accurately replicate the variability of clinical occlusal contacts. Therefore, future work should incorporate multiple contact sites or patient-specific occlusal contact mapping and dynamic loading cycles to better simulate clinical mastication. 40 Moreover, static loading underestimates true functional stresses and fails to capture cyclic fatigue damage, which has been shown to cause longitudinal dentinal cracks under repeated loading. It also omits the weakening impact of cyclic stress on adhesive interfaces. 7 41

## Conclusion

This study indicates that increasing cusp inclination by up to 10 degrees during endocrown fabrication may improve biomechanical performance in molars with limited remaining coronal tooth structure (≤ 1.5 mm above the CEJ). Such adjustment will enhance stress distribution without increasing shear stress at the adhesive interface. The findings support a customized occlusal design tailored to the residual anatomy; however, modifications should be kept within clinically acceptable limits that preserve functional occlusal anatomy, intercuspal stability, and esthetic harmony. While the study's strength lies in its controlled FEA approach, limitations include idealized environment and material assumptions. Further research under more clinically relevant conditions is recommended.
